# Acute Myopericarditis Associated with Tickborne *Rickettsia sibirica mongolitimonae*

**DOI:** 10.3201/eid2312.170293

**Published:** 2017-12

**Authors:** Pablo Revilla-Martí, Álvaro Cecilio-Irazola, Jara Gayán-Ordás, Isabel Sanjoaquín-Conde, Jose Antonio Linares-Vicente, José A. Oteo

**Affiliations:** Hospital Clínico Universitario Lozano Blesa, Zaragoza, Spain (P. Revilla-Martí, A. Cecilio-Irazola, J. Gayán-Ordás, I. Sanjoaquín-Conde, J.A. Linares-Vicente);; Centro de Investigación Biomédica de La Rioja, Logroño, Spain (J.A. Oteo)

**Keywords:** Rickettsia, ticks, tickborne diseases, bacteria, myocarditis, vector-borne infections, Spain, *Rickettsia sibirica mongolitimonae*

## Abstract

We report an unusual case of myopericarditis caused by *Rickettsia sibirica mongolitimonae*. Because of increasing reports of *Rickettsia* spp. as etiologic agents of acute myopericarditis and the ease and success with which it was treated in the patient reported here, rickettsial infection should be included in the differential diagnosis for myopericarditis.

Myopericarditis is a primarily pericardial inflammatory syndrome occurring when clinical diagnostic criteria for pericarditis are satisfied and concurrent mild myocardial involvement is documented by elevated biomarkers of myocardial damage (i.e., increased troponins). Limited clinical data on the causes of myopericarditis suggest that viral infections are among the most common causes in developed countries, although the list of agents is increasing. We identified an unusual case of myopericarditis caused by *Rickettsia sibirica mongolitimonae*, an emerging pathogen in southern Europe with a broad clinical spectrum (*1*).

In September 2016, a 39-year-old man with no remarkable medical history sought care at an emergency department in Spain with acute-onset central chest pain and fever. The previous week, he had hunted in northeastern Spain. Physical examination revealed a systolic blood pressure of 115 mm Hg, heart rate 80 beats/min, peripheral pulse oximetry of 98%, and an axillary temperature of 38.7°C. No murmurs, rales, or gallops were detected on cardiac examination. A necrotic left gluteus eschar and multiple enlarged left inguinal lymph nodes were noted. He had neither lymphangitis nor widespread rash, and his mucous membranes appeared normal. He did not remember tick bites.

An electrocardiogram demonstrated a sinus rhythm with diffuse ST-segment elevation, and a transthoracic echocardiogram showed a normal biventricular ejection fraction with mild pericardial effusion. High-sensitive T troponin level was 575.3 ng/L (reference <14 ng/L), and blood cultures and serologic tests for common viruses were all negative. He was admitted to the hospital, and a cardiac magnetic resonance study performed 48 hours later confirmed the suspected diagnosis of myopericarditis.

Because of the eschar, tickborne-related rickettsiosis was suspected, and ibuprofen (1,800 mg/d) and doxycycline (100 mg every 12 h) were started. After the third day on medical therapy, the patient became afebrile, and the electrocardiographic changes gradually resolved. He was discharged after 12 days. Doxycycline was maintained for 14 days. 

Acute-phase serologic tests yielded negative results for HIV; *Borrelia burgdorferi* sensu lato (chemiluminiscence immunoassay, Liason, Diasorin, Spain); spotted fever group rickettsia (SFGR) (commercial [Focus Diagnostics, Cypress, CA, USA] and in-house tests); and *Francisella tularensis* (in-house microagglutination assay). An eschar swab sample and an eschar biopsy sample were removed under aseptic conditions and sent together with EDTA-treated blood and serum specimens to Spain’s reference center for rickettsioses (Hospital San Pedro–Centro de Investigación Biomédica de La Rioja, Logroño, Spain) for molecular analysis. Samples were tested by PCR for the presence of *Rickettsia* spp. (*ompB*, *ompA*, and *sca 4* genes). Fragments of *ompB* rickettsial genes (285/285 bp) were amplified from the eschar biopsy and swab. The sequences obtained showed 99.8% identity to the corresponding sequences of *R. sibirica mongolitimonae* (GenBank accession no. AF123715).

A convalescent-phase serum specimen collected 7 weeks after hospital discharge was tested by indirect immunofluorescence assay for IgG against SFGR. Commercial (Focus Diagnostics) and in-house *R. conorii* and *R. slovaca* antibody testing showed an IgG of 1:1,024. In-house microagglutination assay results for *F. tularensis* were not reactive.

Myopericarditis, a rare complication of human rickettsiosis, usually occurs with acute infection caused by *R. rickettsii* or *R. conorii*. To our knowledge, there are few reports of a myopericarditis due to SFGR infections (Table) (*2*–*9*), and in PubMed, we found none attributed to *R. sibirica mongolitimonae*.

**Table:** Characteristics of adults previously reported with myopericarditis associated with *Rickettsia* spp. infection*

Characteristic	Patient 1 (*2*)	Patient 2 (*3*)	Patient 3 (*4*)	Patient 4 (*5*)	Patient 5 (*6*)	Patient 6 (*7*)	Patient 7 (*8*)	Patient 8 (*9*)
Age, y/sex	28/M	52/F	74/F	35/M	25/F	54/M	Unk/unk	26/M
Country	Spain	Australia	South Africa	South Africa	United States	United States	Sri Lanka	United States
Signs and symptoms								
Fever	No	Yes	Yes	Yes	Yes	Yes	Yes	Yes
Rash	No	Yes	Yes	Yes	Yes	No	Unk	Yes
Adenopathy	Yes	No	Yes	No	Unk	Unk	Unk	Unk
Lymphangitis	Yes	No	Yes	Yes	Unk	Unk	Unk	Unk
Headache	Yes	Yes	Yes	No	Unk	Yes	Unk	Unk
Myalgia	No	Yes	No	No	Unk	Yes	Unk	Unk
Chest pain	Yes	Yes	Yes	Yes	Unk	Yes	Unk	Unk
Heart failure	No	Yes	Yes	No	Unk	No	Unk	Unk
Eschar	Yes; neck	No	Yes; unk	Yes; abdomen	Unk	Unk	No	No
Organism	*R. slovaca*	*R. australis*	*R. africae*	*R. africae*	*R. rickettsii*	*R. rickettsii*	*R. conorii*	*R. rickettsii*
LVD	No	Yes	Unk	No	Yes	Yes	Unk	Yes
PE	No	No	Unk	No	Yes	No	Unk	No
Treatment	Doxycycline	Tigecycline, doxycycline	Doxycycline	Doxycycline	Unk	Doxycycline	Doxycycline	Doxycycline

*LVD, left ventricular dysfunction; PE, pericardial effusion; unk, unknown.

*R. sibirica mongolitimonae* is an intracellular bacterium that was first reported as a human pathogen in 1996; since then, several cases have been reported from France, Portugal, Greece, and Spain showing seasonal variations with predominance during spring and summer (*1*). Clinical manifestations include fever with or without rash, myalgia, and headache. A characteristic rope-like lymphangitis from the eschar to the draining lymph node is evident in one third of patients (*1*).

Rickettsiosis is commonly diagnosed on the basis of serologic testing, although use of molecular tools or cell culture on a skin biopsy specimen from an eschar is one of the best methods to identify *Rickettsia* spp. Swabbing an eschar is painless, and its results are similar to skin biopsy samples by molecular tools. In the patient we reported, the swab sample from the eschar was useful for rickettsial diagnosis (*10*). Negative test results for other agents and the clinical response to doxycycline strongly supported the diagnosis of acute myopericarditis associated with *R. sibirica mongolitimonae*. Because of increasing reports of different species of *Rickettsia* involved as etiologic agents of acute myopericarditis and the ease and success with which this infection was treated, we strongly recommend including rickettsial infection in the differential diagnosis in the adequate epidemiology context.
